# Tumor glucose reprogramming suppresses cuproptosis: A review

**DOI:** 10.17305/bb.2025.12751

**Published:** 2025-08-06

**Authors:** Xiao-Hang Song, Yi-Hang Ding, Jing-Song Chen

**Affiliations:** 1 Department of Gastrointestinal Surgery, The First Affiliated Hospital of Guangzhou Medical University, Guangzhou, China

**Keywords:** Cuproptosis, cell death, glucose metabolic reprogramming, tumor

## Abstract

Cuproptosis is a copper-dependent form of regulated cell death that begins when ferredoxin 1 (FDX1) reduces Cu^2+^ to Cu^1+^, allowing the ion to bind lipoylated enzymes of the tricarboxylic acid (TCA) cycle, drive protein aggregation, dismantle iron–sulphur clusters and trigger fatal proteotoxic stress. Most tumors, despite accumulating copper, evade this fate through glucose-metabolic rewiring. First, oncogenic stabilization of hypoxia-inducible factor-1 alpha (HIF-1α) and MYC increases pyruvate dehydrogenase kinase (PDK) activity, which phosphorylates and inactivates the pyruvate dehydrogenase complex (PDC), shrinking the lipoylated target pool in mitochondria and cutting the feed into the TCA cycle. Second, glycolytic signalling suppresses cuproptosis-promoting genes such as FDX1 and dihydrolipoamide S-acetyltransferase while inducing the negative regulator glutaminase (GLS), further lowering copper sensitivity. Third, diversion of glycolytic intermediates into the pentose-phosphate pathway (PPP) supplies abundant nicotinamide adenine dinucleotide phosphate (NADPH), whereas enhanced glutamine (Gln) catabolism furnishes glutamate (Glu); together these fuels expand reduced glutathione (GSH) and metallothionein (MT) pools that chelate Cu^1+^ and quench reactive oxygen species exactly where cuproptosis is executed. Consequently, glycolysis-dependent cancer cells are far less sensitive to copper-ionophore drugs such as elesclomol or disulfiram than respiration-dependent counterparts, and clinical datasets consistently link high PDK and low PDC-subunit expression with poor prognosis. These insights highlight rational combination strategies: re-activating the TCA cycle with PDK inhibitors, draining PPP- or GLS-driven NADPH/GSH supply, and concurrently delivering copper ionophores could reopen the cuproptotic trap in tumors. Validating such approaches in vivo, charting upstream regulators of FDX1 and mapping crosstalk between cuproptosis and other lethal programmes remain key steps toward exploiting this copper-centred vulnerability in cancer therapy.

## Introduction

Copper is a trace element that serves as a cofactor for numerous enzymes [1, 2]. However, elevated levels of copper can result in cellular apoptosis. Recent research has elucidated the specific mechanism underlying copper-induced cell death, termed cuproptosis. This novel form of cell death is distinct from previously recognized mechanisms and is closely linked to mitochondrial respiration and the lipoylated proteins involved in the mitochondrial tricarboxylic acid (TCA) cycle [3]. Tumor glucose metabolic reprogramming is a critical hallmark of cancer, encompassing aerobic glycolysis and the highly active pentose phosphate pathway (PPP). Aerobic glycolysis rapidly generates adenosine triphosphate (ATP) for tumor cells, while the lactic acid produced during this process contributes to an acidic tumor microenvironment that facilitates tumor migration [4–7]. Additionally, the PPP pathway has been shown to supply essential substrates for tumor proliferation and to produce nicotinamide adenine dinucleotide phosphate (NADPH), a key molecule involved in cellular proliferation [8]. Cuproptosis is intricately linked to the reprogramming of glucose metabolism in tumors. Genes associated with cuproptosis are involved in the aerobic oxidation of glucose and are frequently downregulated during tumor glucose metabolic reprogramming. Furthermore, cells reliant on glycolysis exhibit resistance to cuproptosis compared to those dependent on mitochondrial respiration. The highly active PPP produces NADPH, which aids in the reduction of oxidized glutathione (GSSG) to its reduced form (GSH), thereby enhancing cellular GSH levels. This process serves as a crucial mechanism for inhibiting cuproptosis through copper chelation [3, 8]. Consequently, the reprogramming of glucose metabolism may serve as an effective strategy for tumors to mitigate cuproptosis. This review examines the mechanisms underlying cuproptosis and the significant relationship between cuproptosis and tumor glucose metabolic reprogramming, providing potential avenues for future research in this area.

### Literature search and selection

A comprehensive literature search was conducted to identify relevant studies published up to June 2025. Databases including PubMed, Web of Science, Scopus, and Google Scholar were systematically queried using the following core keywords and their combinations: cuproptosis, copper metabolism, glucose metabolic reprogramming, Warburg effect, pentose PPP, glycolysis, tumor metabolism, PDH complex, FDX1, DLAT, glutathione, GSH, PDK, PKM2, LDHA, HK2, and GLUT.

Inclusion criteria included: (1) Original research articles and reviews focusing on the molecular mechanisms of cuproptosis and glucose metabolism in tumors. (2) Studies elucidating the interplay between copper-induced cell death and metabolic pathways. (3) Publications in English with full-text availability.

Exclusion criteria were: (1) Articles unrelated to tumor metabolism or copper biology. (2) Studies based solely on non-mammalian models or non-cancerous contexts. (3) Abstracts, conference proceedings, or non-peer-reviewed works.

The initial screening of titles and abstracts was followed by a full-text review of eligible articles. Key references were further expanded through citation tracking, with a focus on high-impact studies published within the past five years, supplemented by seminal historical papers.

### Copper homeostasis

**Figure 1.**
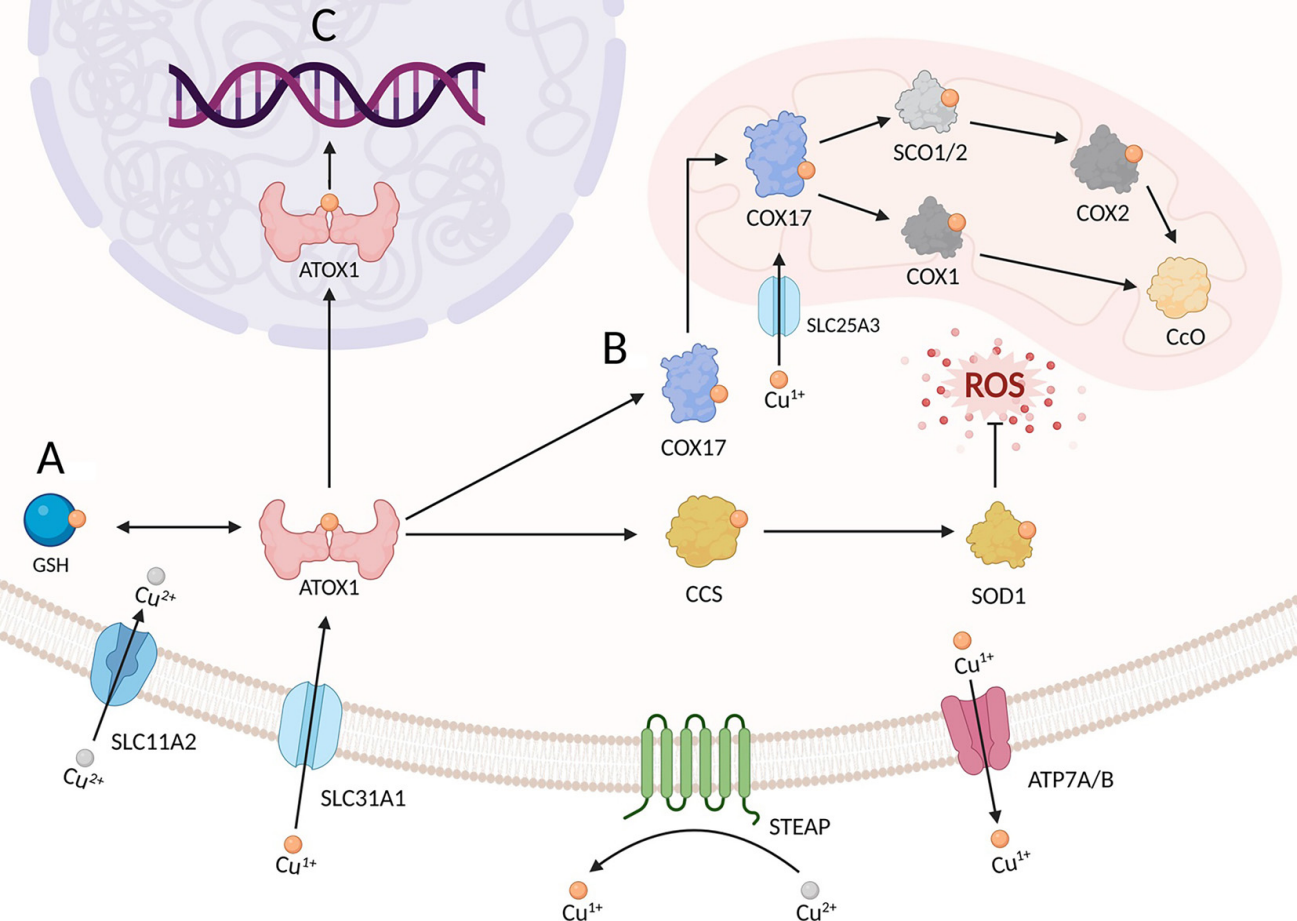
**Summary of copper metabolism**. (A) Extracellular Cu^2+^ is first reduced to Cu^1+^ by STEAP reductases, followed by primary uptake via the SLC31A1 transporter. Concurrently, SLC11A2 mediates auxiliary Cu^2+^ transport. Within the cytosol, Cu^1^+^^ binds either to ATOX1—which delivers copper to CCS for loading onto SOD1 to enable ROS scavenging—or to glutathione (GSH) forming complexes. Cellular copper efflux is ultimately mediated by ATP7A/B transporters. (B) Cu^1+^ enters mitochondria via COX17/SLC25A3. COX17 delivers copper to SCO1/SCO2 for Cu incorporation into CcO’s COX1 subunit, enabling CcO assembly and ETC function. (C) ATOX1 transports Cu^1+^ into the nucleus to regulate gene expression.

Copper (Cu) is an essential trace element in the human body, with an average content of approximately 110 mg [9]. The body acquires copper ions from various dietary sources, including meats, grains, water, and vegetables. Additionally, animal offal and nuts serve as significant sources of copper [9]. This metal is widely distributed throughout the body, with an estimated concentration of 10 mg in the liver, 1 µg/mL in plasma, and 28 mg in muscle tissue, while bone exhibits the highest concentration at approximately 46 mg [9–11]. In biological systems, dietary copper predominantly exists as Cu(II), whereas the essential form for physiological functions is Cu(I) [11]. The process of copper absorption is a complex physiological phenomenon primarily occurring in the epithelial cells of the small intestine. The initial reduction of Cu(II) to Cu(I) is facilitated by six-transmembrane epithelial antigens of the prostate 2–4 (STEAP2–4) [12, 13]. Once absorbed, copper binds to copper chaperone proteins in the cytoplasm to perform its physiological roles. The antioxidant 1 copper chaperone (ATOX1) is crucial in the copper trafficking pathway, where it can accept Cu(I) from SLC31A1, thioltransferase-1 (GRX1), and GSH-binding copper [14–17]. Subsequently, copper bound to ATOX1 is transported to ATPase copper transporting Alpha (ATP7A) on the basolateral membrane, where it is either released into the plasma or directed to the nucleus [10, 18, 19]. In the plasma, copper is present in various protein-bound forms, the most prevalent being ceruloplasmin (CP), along with alpha-2-macroglobulin (A2M) and superoxide dismutase 3 (SOD3) [20]. Transhepatic metabolism is the primary pathway for copper, with the liver serving as a key organ for copper storage after binding to metallothionein-1/2A (MT1/2) [21, 22]. The ATPase copper-transporting beta (ATP7B) enzyme is predominantly expressed in the liver, specifically localized to the Golgi apparatus, where it plays a critical role in copper metabolism. When intracellular copper levels rise, ATP7B is translocated to the surface of intrahepatic bile ducts, facilitating the excretion of copper into the bile [21, 23]. Copper chaperone for superoxide dismutase (CCS) is a crucial copper chaperone protein located in the cytoplasm. In vitro studies demonstrate that CCS can interact with ATOX1 to facilitate copper delivery [19]. Intracellularly, CCS transports copper to superoxide dismutase 1 (SOD1) and facilitates the insertion of disulfide bonds, which are essential for SOD1 activation [20]. Once activated, SOD1 effectively scavenges reactive oxygen species (ROS) *in vivo* through the reduction of Cu(I) [24]. Cytochrome C oxidase copper chaperone COX17 (COX17) is located in mitochondria, where it serves as a cofactor in the antioxidative stress response of SOD1 and facilitates the entry of copper into the oxidative respiratory chain. The solute carrier family 25 member 3 (SLC25A3) and COX17 work together to translocate copper to the mitochondria [2, 25]. The binding of copper to COX17 is crucial for maintaining the integrity of the mitochondrial electron transport chain. COX17 transports copper to cytochrome C oxidase copper chaperone COX11 (COX11), where copper, in conjunction with cytochrome C oxidase copper chaperone COX19 (COX19), is reduced to maintain Cu(I) and promote copper binding to the Cu(B) site of cytochrome C oxidase copper chaperone COX1 (COX1) [17, 25]. Conversely, COX17 serves as a copper donor for SCO1/2 and is translocated to the Cu(A) binding site of COX2 with the assistance of COA6 [17]. Ultimately, COX1 and COX2 are essential for the synthesis of cytochrome c oxidase (CcO) (Figure 1) [26, 27].

### The mechanism of cuproptosis

Proper regulation of copper homeostasis is crucial for biological growth, development, and normal physiological function. A deficiency of intracellular copper can lead to severe developmental delays and morphological abnormalities [27, 28]. Conversely, copper overload has been linked to cell death, as evidenced by the presence of copper and other elemental residues in the nuclei of hepatocytes in male rats subjected to a high copper diet (1500 ppm). These residues are strongly associated with subsequent hepatocyte death [29]. Additionally, Keswani et al. demonstrated that excess copper induces cell cycle arrest and apoptosis in spleen and thymus cells, indicating that copper-induced cell death may be a widespread phenomenon across various cell types [3]. However, the precise mechanisms through which copper induces cell death remain unclear.

**Figure 2.**
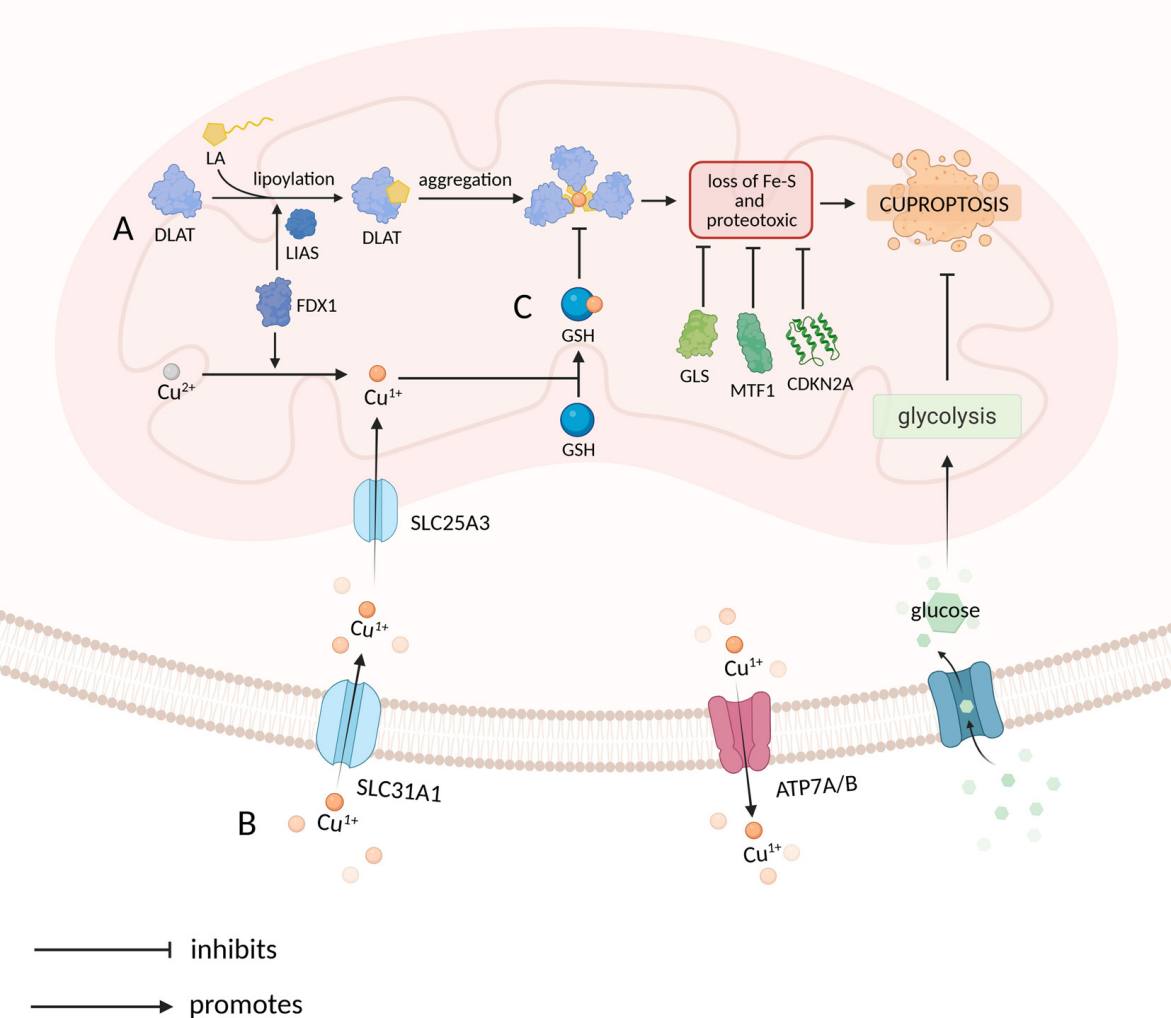
**Mechanism and regulation of cuproptosis**. (A) The major pathway of cuproptosis. FDX1 is the upstream regulator of cuproptosis and promotes DLAT lipoylation through direct binding to LIAS. The lipoylation of DLAT binds to Cu^1+^, resulting in aggregation. This process leads to proteotoxic stress, loss of Fe-S and cell death. (B) Copper in the pathway of cuproptosis. Cu^1+^ is transported into the cytoplasm via SLC31A1 and into the mitochondria via SLC25A3. In mitochondria, Cu^2+^ can be reduced to Cu^1+^ by FDX1. Cu^1+^ binds to the lipoylation of DLAT to facilitate the process of cuproptosis and can also be bound by GSH. (C) Factors negatively associated with cuproptosis. The green labels represent factors that are negatively associated with cuproptosis. These include genes that are negatively associated with cuproptosis (GLS, CDKN2A, and MTF1), glycolysis, GSH, and ATP7A/B.

In 2022, Tsvetkov et al. conducted a study that elucidated the mechanism of copper-induced cell death, termed cuproptosis. This form of cell death was observed exclusively with copper-bound ionophores, such as elesclomol and disulfiram, thereby confirming that cuproptosis is specifically initiated by intracellular copper overload mediated through these carriers, rather than by ionophores alone or by extracellular copper. This finding distinguishes cuproptosis from other forms of cell death [3]. Cuproptosis is mechanistically characterized by three defining features: (1) strict dependence on copper, (2) a requirement for functional mitochondrial respiration, and (3) multilayered regulatory mechanisms. A specific mechanism underlying cuproptosis has been identified involving copper overload, which promotes the aggregation and functional impairment of lipoylation proteins by binding to them within the TCA cycle. This process ultimately results in the loss of iron–sulfur (Fe–S) cluster proteins, leading to proteotoxic stress and cell death. A total of ten genes associated with cuproptosis have been identified, comprising seven cuproptosis-positive genes and three cuproptosis-negative genes. The cuproptosis-positive genes include ferredoxin 1 (FDX1), components of the lipoic acid pathway—lipoyltransferase 1 (LIPT1), lipoic acid synthetase (LIAS), and dihydrolipoamide dehydrogenase (DLD)—as well as subunits of the pyruvate dehydrogenase (PDH) complex: dihydrolipoamide S-acetyltransferase (DLAT), pyruvate dehydrogenase E1 subunit alpha 1 (PDHA1), and pyruvate dehydrogenase E1 subunit beta (PDHB) [3]. The cuproptosis-negative genes include metal regulatory transcription factor 1 (MTF1), glutaminase (GLS), and cyclin-dependent kinase inhibitor 2A(CDKN2A) [3]. FDX1 serves as the upstream regulator of cuproptosis by promoting the lipoylation of DLAT through its direct interaction with LIAS. Additionally, FDX1 facilitates the reduction of Cu(II) to the more cytotoxic Cu(I) [3, 30]. The knockout of FDX1 inhibits protein lipoylation and prevents cuproptosis [3]. Lipoic acid-dependent protein lipoylation is a highly conserved post-translational modification occurring on four key enzymes: DLAT, dihydrolipoamide S-succinyltransferase (DLST), glycine cleavage system protein H (GCSH), and dihydrolipoamide branched-chain transacylase E2 (DBT). These enzymes are integral to metabolic complexes that regulate the entry of carbon into the TCA cycle [3]. The lipoylation of DLAT is essential for its binding to copper, as copper directly interacts with lipoylated DLAT, leading to its aggregation. This aggregation induces proteotoxic stress and results in the loss of Fe–S cluster proteins [3]. Furthermore, abnormalities in SLC31A1, ATP7A, and ATP7B have been linked to dysregulated copper homeostasis. Dysregulation of copper homeostasis ultimately leads to cell death via a mechanism analogous to cuproptosis [3]. Furthermore, several factors have been identified that negatively correlate with cuproptosis, including hypoxic conditions, glycolysis-dependent cells, and those rich in glutathione (GSH) [3]. Tsvetkov et al. [3] established cuproptosis as a distinct form of cell death mediated by the targeting of lipoylated TCA cycle proteins by copper (Figure 2). This discovery has significant implications for understanding disorders of copper homeostasis, such as Wilson’s and Menke’s diseases [31, 32], and has opened new therapeutic avenues for cancer and metabolic diseases [33, 34]. Notably, recent translational studies have validated these mechanistic insights, demonstrating that LIPT1—a core component of the lipoylation pathway—functions as both a prognostic biomarker and a therapeutic target in non-small cell lung cancer (NSCLC). Tumor-specific downregulation of LIPT1 not only impairs protein lipoylation but also suppresses ATOX1 expression, thereby establishing a dual resistance mechanism against cuproptosis [35]. Nevertheless, several critical aspects of cuproptosis remain to be elucidated: (1) the causal relationship between the loss of Fe–S cluster proteins and the execution of cuproptosis, (2) the upstream regulators that govern FDX1 expression and activity, and (3) the context-dependent functions of other cuproptosis-related genes.

### Prospective on mechanisms underlying the regulation of cuproptosis by tumor glucose metabolic reprogramming

In the 1920s, Otto Warburg observed that tumors preferentially utilize glycolysis for energy production instead of oxidative phosphorylation, even in aerobic conditions. This phenomenon, known as the Warburg effect or aerobic glycolysis, results in less efficient ATP generation compared to oxidative phosphorylation. However, aerobic glycolysis allows for rapid ATP production to meet the energy demands of tumor metabolism [4]. Additionally, the lactic acid generated during aerobic glycolysis contributes to the formation of an acidic tumor microenvironment, which has been shown to enhance tumor proliferation, invasion, and migration [5–7]. Moreover, the activity of the PPP is significantly elevated in tumors [8, 36]. This increased PPP activity provides essential substrates for tumor growth, while its high NADPH production helps mitigate oxidative stress within the tumor. Finally, the decreased entry of glucose into the TCA cycle during tumor metabolic reprogramming is associated with enhanced Gln utilization [37]. Copper serves as a cofactor for various enzymes and plays a significant role in tumor development. Elevated serum copper concentrations have been reported in patients with multiple malignant tumors, including breast, lung, liver, and colorectal cancers (CRCs) [38–43]. Copper is involved in the activation of several tumor-related signaling pathways. In hepatocellular carcinoma, copper accumulation activates hypoxia-inducible factor 1 subunit alpha (HIF-1α) [44], which is strongly associated with hepatocellular carcinoma growth, angiogenesis, metastasis, and drug resistance [45–47]. Additionally, copper mediates tumor immune escape by promoting the expression of programmed cell death 1 ligand 1 (PD-L1) in neuroblastoma, in conjunction with interferon gamma (IFNγ) [48]. Copper also facilitates ligand-independent activation of receptor tyrosine kinases (RTKs), resulting in downstream phosphorylation of phosphoinositide 3-kinase (PI3K) and activation of AKT, which promote tumor cell growth and migration in lung, prostate, and breast cancer cell lines [49, 50]. The reliance of tumors on copper fosters an environment conducive to cuproptosis; however, the evasion of cuproptosis by tumors may be intricately linked to tumor glucose metabolic reprogramming. Investigating the relationship between cuproptosis and tumor glucose metabolic reprogramming is essential for future research and potential therapeutic applications of cuproptosis in oncology.

### Tumor glucose metabolic reprogramming inhibits cuproptosis by increasing dependence on aerobic glycolysis

Cells exhibiting different modalities of glucose metabolism show varying sensitivities to cuproptosis. Tsvetkov et al. [3] demonstrated that cells reliant on mitochondrial respiration exhibit significantly greater sensitivity to copper ionophore-induced cuproptosis compared to glycolytic cells, as quantified by dose-response assays. The dependence on aerobic glycolysis, a prevalent metabolic pathway in tumors, may provide a selective advantage that enables tumor cells to evade cuproptosis [51, 52]. Alterations in the expression of numerous genes associated with glucose metabolism are common in tumors and are linked to the promotion of aerobic glycolysis. To enhance glucose uptake, tumors upregulate the expression of various members of the solute carrier family 2 (GLUTs). In pancreatic cancer, forkhead box D1 (FOXD1) enhances GLUT1 expression by facilitating its transcription and inhibiting its degradation. The overexpression of GLUT1 has been shown to promote aerobic glycolysis, as well as the proliferation, invasion, and metastasis of pancreatic cancer [53]. In pancreatic cancer, mutations in K-Ras lead to the upregulation of GLUT2 expression, thereby promoting aerobic glycolysis [54]. A prior study indicated that inhibiting aerobic glycolysis in tumors can be effectively achieved using glycolysis inhibitors. Conversely, the expression of GLUT1 and GLUT3 in a glucose-deficient tumor environment is sufficient to sustain aerobic glycolysis [55]. During tumor glucose metabolic reprogramming, the expression and activity of various proteins within the glycolytic pathway are altered. Hexokinases (HKs) serve as the first key enzymes in glycolysis, catalyzing the conversion of glucose to glucose-6-phosphate. There are five distinct hexokinase phenotypes: HK1, HK2, HK3, HK4, and hexokinase domain-containing protein-1 (HKDC1). HK1 is relatively conserved across various tissues under different physiological conditions. In contrast, HK2 and HKDC1 are frequently overexpressed in numerous tumors and are associated with poor prognostic outcomes [56].

**Figure 3.**
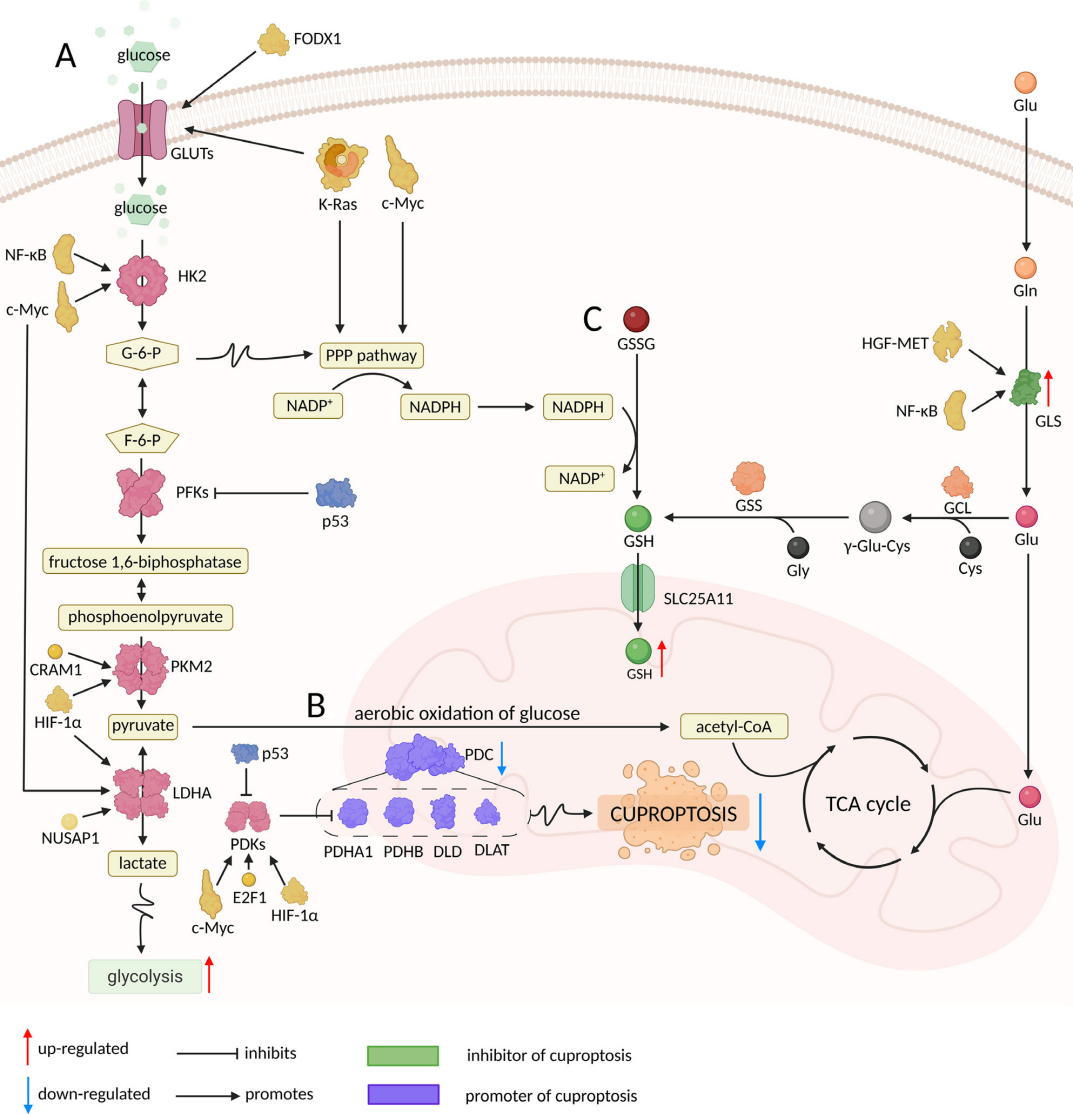
**Comprehensive overview of the potential mechanisms through which tumor glucose metabolic reprogramming regulates cuproptosis**. (A) Tumors promote aerobic glycolysis by regulating glycolysis-related genes, which subsequently inhibits cuproptosis; (B) Tumor glucose metabolic reprogramming inhibits cuproptosis-related genes via PDKs, subsequently suppressing cuproptosis; and (C) PPP sustains reduced GSH via NADPH for Cu^+^-chelation, depleting mitochondrial labile copper to block cuproptosis.

Multiple tumor-related pathways are activated to enhance the expression of hexokinase 2 (HK2). In hepatitis B virus (HBV)-associated hepatocellular carcinoma, the activation of the nuclear factor kappa-B (NF-κB)/p65/HK2 signaling pathway induces aerobic glycolysis in tumors, thereby facilitating the development of hepatocellular carcinoma [57]. Additionally, small nucleolar RNA host gene 26 (SNHG26) interacts with nucleolin (NCL) to elevate the expression of the Myc proto-oncogene protein (c-MYC), which further promotes aerobic glycolysis and tumor progression by increasing HK2 expression [58]. Moreover, HK2 gene expression has been found to correlate strongly with aerobic glycolysis and plays a significant role in the progression of breast and lung cancers [59, 60]. Phosphofructokinases (PFKs) serve as the second key enzyme in the glycolytic pathway, catalyzing the conversion of fructose 6-phosphate (F-6-P) to fructose 1,6-bisphosphate (F-1,6-BP) [61]. Numerous studies have demonstrated that PFK plays a critical role in the regulation of aerobic glycolysis. In CRC, the activation of mutant tumor protein p53 inhibits the progression of breast cancer by downregulating PFK, which in turn reduces tumor proliferation, migration, and extracellular lactate levels [62]. Similarly, in head and neck squamous cell carcinoma, the inhibition of PFKFB3 resulted in a notable decrease in glucose uptake, lactate production, and ATP generation [63]. Pyruvate kinase (PK), the final key enzyme in the glycolytic pathway, catalyzes the production of pyruvate [64]. PK consists of four isoforms (PKM1, PKM2, PKR, and PKL), with PKM2 being the most abundantly expressed in various tissues and closely associated with aerobic glycolysis and tumorigenesis [64]. In breast cancer, the methylation of coactivator-associated arginine methyltransferase 1 (CARM1) modifies PKM2, leading to its conversion from a dimer to a tetramer, which enhances PKM2 activity. The highly active form of PKM2 significantly promotes aerobic glycolysis in breast cancer [65]. In hepatocellular carcinoma, the activation of the HIF-1α/peroxisome proliferator-activated receptor gamma (PPAR-γ)/PKM2 axis leads to the upregulation of PKM2 expression, subsequently promoting aerobic glycolysis in tumors. This metabolic shift contributes to the development of drug resistance in hepatocellular carcinoma [66]. The conversion of pyruvate to lactic acid, catalyzed by lactate dehydrogenase (LDHA), represents the final step in aerobic glycolysis. LDH exists in six distinct isoforms, with LDH1-5 comprising various combinations of LDHA and LDHB, while LDH6 corresponds to LDHC [67]. Among these isoforms, LDHA exhibits a higher affinity for pyruvate, facilitating its conversion to lactate [67]. LDHA is notably overexpressed in numerous tumors and is closely linked to tumor-associated aerobic glycolysis. In CRC, the enzyme methyltransferase 3 (METTL3) enhances aerobic glycolysis by promoting the translation of LDHA mRNA via HIF-1α mRNA, as well as by methylating the coding sequence of LDHA and recruiting the YTH N6-Methyladenosine RNA binding protein F1 (YTHDF1) [68]. The elevated expression of lactate dehydrogenase A (LDHA) in pancreatic cancer is closely linked to increased levels of aerobic glycolysis. Nucleolar and spindle-associated protein 1 (NUSAP1) has been shown to enhance LDHA expression and promote aerobic glycolysis in pancreatic cancer by directly binding to c-Myc and HIF-1α [69]. Furthermore, Aurora-A has been implicated in the stimulation of glycolysis through the phosphorylation of lactate dehydrogenase B (LDHB), indicating that tumor cells may modify their metabolic state by regulating the balance between LDHA and LDHB [70]. Collectively, tumor glucose metabolic reprogramming alters the expression and activity of numerous enzymes involved in the glycolytic pathway, including glucose transporters (GLUTs), HKs, PFKs, pyruvate kinases (PKMs), and LDHs (Figure 3A). This shift toward aerobic glycolysis is essential for tumors to meet their metabolic demands and adapt to their surrounding growth environment. Additionally, this altered metabolic state reduces the flow of pyruvate into the TCA cycle, thereby diminishing the tumor’s reliance on oxidative phosphorylation and increasing its dependence on glycolysis. The tumor’s over-reliance on aerobic glycolysis also subtly mitigates the effects of cuproptosis.

### Tumor glucose metabolic reprogramming and cuproptosis-related genes

Multiple cuproptosis-related genes are closely associated with the reprogramming of glucose metabolism in tumors. Consequently, changes in tumor glucose metabolic status may directly influence the expression profiles of these cuproptosis-related genes, thereby inhibiting the occurrence of cuproptosis.

The pyruvate dehydrogenase complex (PDC) is a crucial enzyme that catalyzes the conversion of pyruvate to acetyl-CoA, linking it to the TCA cycle. The PDC comprises three components: pyruvate dehydrogenase (E1), dihydrolipoamide acetyltransferase (DLAT), and dihydrolipoamide dehydrogenase (E3). Among these, the DLAT, PDHA1 of the E1 subunit, and PDHB of the E1 subunit are essential genes for cuproptosis [3, 71–73]. During the aerobic oxidation of glucose, pyruvate binds to the E1 subunit in the presence of thiamine pyrophosphate (TPP) and undergoes decarboxylation. This is followed by the lipoylation of DLAT, which couples the E1 and E3 subunits, ultimately leading to the synthesis of acetyl-CoA. The activation of the PDC is critical for both the aerobic metabolism of glucose and the induction of cuproptosis [3]. The activity of PDC in tumor glucose metabolic reprogramming is significantly reduced. Pyruvate dehydrogenase E1 alpha subunit (PDHA1) plays a crucial role in this process, as its inhibition represents a key step in the suppression of PDC. Pyruvate dehydrogenase kinases (PDKs), comprising types PDK1 through PDK4, contribute to the inactivation of PDC by phosphorylating serine residues on PDHA1. In CRC, the non-SMC condensin II complex subunit D3 (NCAPD3) further inhibits PDC and the TCA cycle by elevating E2F1 levels and interacting with E2F1, which is recruited to the promoter regions of the PDK1 and PDK3 genes [74]. The upregulation of HIF-1α is strongly linked to glucose metabolic reprogramming, primarily through the stabilization of HIF-1α, which induces the upregulation of PDK and LDHA [75, 76]. In colorectal and cervical cancers, elevated levels of HIF-1α have been found to enhance the expression of PDK3. The high expression of PDK3 significantly inhibits pyruvate dehydrogenase complex (PDC) activity, thereby facilitating aerobic glycolysis [77, 78]. Additionally, PDK2 plays a crucial role in tumor glucose metabolic reprogramming. The oncogene c-Myc is also implicated in this process, as research indicates that c-Myc promotes the expression of PDK2, which subsequently enhances tumor glucose metabolic reprogramming while inhibiting the TCA cycle in CRC [79]. p53 plays a crucial role in tumor suppression by inhibiting glucose metabolic reprogramming and cell proliferation through the downregulation of PDK2 expression [80]. Elevated levels of PDK4 in high-grade bladder cancer correlate significantly with poor tumor prognosis. Research indicates that the activation of the HIF-1α pathway enhances PDK4 expression, thereby inhibiting PDC activation in bladder cancer and promoting aerobic glycolysis in tumors [81–83].

PDHB is a critical subunit of the E1 enzyme, and its elevated expression is associated with the promotion of cuproptosis. In tumors, high levels of PDHB expression correlate with a favorable prognosis [84–86]. Research indicates that in CRC, miR-146b-5p enhances aerobic glycolysis, tumor growth, invasion, and metastasis by downregulating PDHB expression [87]. Similarly, in ovarian cancer, miR-203 has been shown to facilitate aerobic glycolysis, tumor growth, and migration through the inhibition of PDHB expression [88]. GLS is a negative regulator of cuproptosis and catalyzes the conversion of Gln to Glu [3]. During tumorigenesis, glucose metabolism is reprogrammed, resulting in a decreased reliance on glucose within the TCA cycle. Instead, Gln serves as a crucial carbon source for the TCA cycle to support tumor metabolism and biosynthesis. To meet the increased demand for Gln, GLS expression is significantly upregulated, which correlates strongly with poor prognostic outcomes in tumors [89]. In hepatocellular carcinoma, the aberrant activation of the hepatocyte growth factor (HGF) and hepatocyte growth factor receptor (MET) pathway is closely linked to tumor growth, invasion, and metabolic reprogramming. Activation of this pathway enhances GLS activity while inhibiting pyruvate dehydrogenase complex (PDC) activity, thereby promoting aerobic glycolysis and suppressing cuproptosis in tumors [90]. In leukemia, GLS has been found to facilitate the utilization of Gln as an alternative carbon source for the TCA cycle, following the reprogramming of tumor glucose metabolism. This process is mediated by NF-κB, which enhances GLS expression [91]. In CRC, increased expression of genes associated with aerobic glycolysis—such as GLUT1, HK2, and pyruvate kinase isoform M2 (PKM2)—correlates with elevated GLS levels [92]. Numerous cuproptosis-related genes are altered during tumor glucose metabolic reprogramming, which simultaneously promotes the initiation of this reprogramming and inhibits cuproptosis (see Figure 3B). Investigating the interplay between cuproptosis-related genes and tumor glucose metabolic reprogramming could potentially modify the metabolic state of tumors and induce cuproptosis, thereby suppressing tumor progression. Nevertheless, several cuproptosis-related genes remain to be examined in relation to tumor glucose metabolic reprogramming. Elucidating the mechanisms of cuproptosis and assessing the relationships between cuproptosis-related genes and tumor glucose metabolic reprogramming are crucial for the therapeutic application of cuproptosis in oncology.

### Tumor glucose metabolic reprogramming inhibits cuproptosis by regulating mitochondrial labile copper pool

The PPP is a crucial mechanism involved in the reprogramming of glucose metabolism in tumors. The PPP produces pentose phosphates, which are vital substrates for DNA and RNA synthesis, as well as NADPH, an essential electron donor for biosynthetic reactions and redox balance [3]. GSH plays a significant role in cellular defense against ROS and in detoxification processes. In cells, GSH exists in two states: the thiol-reduced form (GSH) and the disulfide-oxidized form (GSSG). GSH synthesis occurs via two primary pathways: 1) *De novo* synthesis, where cysteine (Cys) and Glu combine to form γ-glutamylcysteine (γ-Glu-Cys), a reaction catalyzed by Glu–Cys ligase (GCL), followed by the addition of glycine (Gly) catalyzed by glutathione synthetase (GSS); and 2) The reduction of GSSG back to GSH, a process that relies directly on NADPH [93]. Importantly, the PPP serves as the primary source of NADPH in cells. Within the PPP, the oxidative branch, initiated by glucose-6-phosphate dehydrogenase (G6PD), directly generates NADPH. The non-oxidative branch, which involves transketolase (TKT) and transaldolase, primarily functions to interconvert sugar phosphates, thereby replenishing glycolytic intermediates such as fructose-6-phosphate and glyceraldehyde-3-phosphate to maintain the flux through the NADPH-producing oxidative branch [93, 99]. GSH levels are elevated in various tumors and are closely linked to cellular proliferation, metastasis, and chemoresistance. Notably, GSH functions as a principal intracellular copper chelator, directly inhibiting cuproptosis [3, 93]. The subcellular compartmentalization of GSH is crucial for its role in copper detoxification. While the cytosol harbors the majority of cellular GSH, the mitochondrial pool (mGSH), imported through specific carriers such as SLC25A11 (the 2-oxoglutarate carrier), is essential for neutralizing copper ions at the site of cuproptosis within the mitochondrial matrix [94, 95]. Limitations in the transport capacity of SLC25A11 may restrict mGSH availability, potentially diminishing copper chelation efficiency in mitochondria despite elevated cytosolic GSH levels [94, 95]. Gln serves as a crucial substrate that provides Glu for the de novo synthesis of glutathione (GSH). Tumors demonstrate a heightened utilization of Gln, and inhibiting Gln metabolism impairs tumor aerobic glycolysis, disrupts the TME, and suppresses tumor growth [96]. The oncogene c-Myc, known for its role in promoting aerobic glycolysis, also enhances Gln uptake. Elevated expression of c-Myc facilitates the entry of Gln into the TCA cycle and supports GSH synthesis by supplying Glu [97]. Additionally, Gln metabolites contribute to NADPH production through mechanisms such as the malic enzyme, which is essential for reducing GSSG back to GSH [93, 98]. It is important to note that the PPP remains the primary source of cellular NADPH. Key pathways involved in aerobic glycolysis are interconnected with the regulation of the PPP. For example, the oncogenic k-Ras in pancreatic ductal adenocarcinoma stimulates glycolysis and redirects glycolytic intermediates into the PPP [99]. In CRC, the aberrant activation of both aerobic glycolysis and the PPP promotes tumor progression while reducing oxidative stress and apoptosis, likely mediated by increased levels of NADPH and, consequently, GSH [100].

In conclusion, the reprogramming of tumor glucose metabolism enhances the flux through the PPP, particularly its oxidative branch, facilitating the generation of abundant NADPH. This NADPH is crucial for maintaining GSH in its reduced, active form by reducing oxidized glutathione (GSSG). Concurrently, this metabolic reprogramming increases tumor uptake of Gln, providing the substrate Glu and indirectly supplying Cys through the transsulfuration pathway for de novo GSH synthesis. The resulting elevated levels of GSH in tumors scavenge ROS and, importantly, act as a copper chelator, inhibiting cuproptosis. However, the efficacy of this protective mechanism at the mitochondrial execution site is critically dependent on the availability of mitochondrial GSH (mGSH), which may be limited by transport constraints [94, 95] (Figure 3C). Beyond GSH, metallothioneins (MTs) also play a synergistic role in copper detoxification. NADPH derived from tumor glycolysis maintains MTs in a reduced state, thereby enhancing their copper-binding capacity [101]. Notably, hypoxia-inducible factor 1-alpha (HIF-1α) mediated suppression of MTs in hypoxic tumor microenvironments may temporarily render cells susceptible to copper overload; however, this vulnerability is countered by the upregulation of GSH and redirection of glycolytic flux. This dual system of MTs and GSH exemplifies the metabolic plasticity that tumors employ to evade copper toxicity.

### Limitations and future directions

While this review discusses how glucose metabolic reprogramming may suppress cuproptosis in tumors, key limitations exist. First, most evidence comes from *in vitro* studies, requiring validation in animal models and clinical samples [3, 52]. Second, copper’s role in cancer is complex—while generally tumor-promoting, it can also suppress tumors in certain contexts [28, 48]. Future work should clarify these opposing effects. Additionally, how cuproptosis interacts with other cell death pathways like ferroptosis needs exploration [33, 93]. Finally, tumor metabolic flexibility may limit copper-targeting therapies [55, 99].

## Conclusion

As an essential trace element, excess copper can lead to cellular death as well. Tsvetkov et al.’s study revealed the specific mechanism by which copper causes cell death. Cuproptosis represents a novel form of cell death, and cuproptosis-related genes are strongly associated with glucose metabolism. Glycolysis-dependent cells demonstrate resistance to cuproptosis. Cuproptosis may be an effective strategy to anti-tumor.

Tumors are a serious threat to human health, the existing medical treatments for tumors are very limited. Tumorigenesis is influenced by a multitude of factors, with tumor glucose metabolic reprogramming representing one of the hallmarks of tumors. Tumor glucose metabolic reprogramming promotes aerobic glycolysis to accelerate energy supply and alter the tumor microenvironment. The highly active PPP provides sufficient ribose and NADPH to the tumor.

The glucose metabolic reprogramming results in tumor dependence on glycolysis and alters the activity of several cuproptosis-related genes. Tumor glucose metabolic reprogramming also stimulates the activity of the PPP, the product of which, NADPH, is an essential factor in the reduction of GSSH to GSH. Finally, the dependence of tumors on Gln resulting from tumor glucose metabolic reprogramming is also a substrate for GSH synthesis. In conclusion, tumor glucose metabolic reprogramming collectively inhibits cuproptosis by promoting aerobic glycolysis, increasing GSH synthesis, and altering the activity of cuproptosis-related genes. Therefore, inhibition of tumor glucose metabolic reprogramming may be an effective strategy to promote the onset of tumor cuproptosis.

## Data Availability

Data and materials related to this work are available upon request.
